# The roles of Notch1 expression in the migration of intrahepatic cholangiocarcinoma

**DOI:** 10.1186/1471-2407-13-244

**Published:** 2013-05-20

**Authors:** Qi Zhou, Yafeng Wang, Baogang Peng, Lijian Liang, Jiaping Li

**Affiliations:** 1 Department of Hepatobiliary Surgery, the First Affiliated Hospital of Sun Yat-sen University, 58 Zhongshan 2nd Road, Guangzhou, Guangdong, 510080, China; 2 Department of Interventional Oncology, the First Affiliated Hospital, Sun Yat-sen University, 58 Zhongshan 2nd Road, Guangzhou, Guangdong 510080, China

**Keywords:** Intrahepatic cholangiocarcinoma, Notch1, Migration

## Abstract

**Background:**

Notch signaling, a critical pathway for tissue development, contributes to tumorigenesis in many tissues; however, the roles of Notch signaling in Intrahepatic Cholangiocarcinoma (ICC) remains unclear. In this study, we evaluated the expression and effects of Notch1 on cell migration in ICC.

**Methods:**

Multiple cellular and molecular approaches were performed including gene transfection, siRNA transfection, RT-PCR, Western blotting, Rac activation assays and immunofluorescence.

**Results:**

We found that Notch1 was up-regulated in ICC tissues and cell lines. The exogenous expression of Notch1 in glioma cells increased their migratory and invasive capacity. Similarly, the suppression of Notch1 expression inactivated Rac1 and inhibited ICC cell migration. Notch1 over expression induced an Epithelial-to-mesenchymal transition (EMT) phenotype that included enhanced expression of α-SMA and Vimentin, loss of E-cadherin expression, morphological changes and cytoskeletal reorganization in ICC cells.

**Conclusion:**

Notch1 may induce a migratory effect in ICC by causing an epithelial-mesenchymal transition and activating Rac1 and could serve as a novel diagnostic and therapeutic target in patients with ICC.

## Background

Intrahepatic Cholangiocarcinoma (ICC) is the second most common subtype of primary hepatobiliary cancer [1,2]. Significant geographic variation exists in the incidence of cholangiocarcinoma, with the highest incidence in East Asia. Despite advances in surgical and medical therapy, the survival rate is still very poor. The primary reason for the poor prognosis is metastasis, which precludes curative surgical resection. Prognosis is dependent on the presence of free margins in resected tissues and the absence of lymph node metastasis [3]. Increased cell invasion and migration are key phenotypic advantages of malignant cells that favor metastasis. Recent studies have shown that tumor metastasis can be regarded as a reactivation of at least some aspects of the embryonic program of the EMT. During EMT, epithelial cells undergo extensive alterations in gene expression to lose apical/basolateral polarity, sever intercellular adhesive junctions, degrade basement membrane components, and become individual, non-polarized, motile and invasive mesenchymal cells [4].

Notch signaling is an ancient cell signaling system that regulates cell fate specification, stem cell maintenance, and the initiation of differentiation in embryonic and postnatal tissues. Four Notch receptors isoforms, namely Notch1, Notch2, Notch3, and Notch4, and five ligands, Jagged 1 and Jagged 2 belonging to the Serrate family and Delta 1, Delta 3, and Delta-like 4 belonging to the Delta family, have been identified in mammals. The pathway is activated through the interaction of a Notch receptor with a Jagged or Delta-like ligand, leading to proteolytic cleavages of the Notch receptor at two distinct sites. This cleavage releases the Notch intracellular domain (ICN), allowing it to enter the nucleus and function as a transcriptional activator. Importantly, the second cleavage is mediated by the gamma secretase complex, and effective inhibition of Notch activation can be achieved by pharmacological inhibition of this proteolytic activity. Notch signaling is known to regulate many cellular processes, including cell proliferation, apoptosis, migration, invasion, and angiogenesis. Notch expression has been reported to be up-regulated in many human malignancies [5]. Interestingly, the function of Notch signaling in tumorigenesis has been shown to be either oncogenic or anti-proliferative [6-8]. In some tumor types, including skin cancer, human hepatocellular carcinoma and small cell lung cancer, Notch signaling has been shown to play anti-tumor roles rather than oncogenic roles [7]. However, most studies have shown that Notch has oncogenic effects in many human carcinomas. In cervical, lung, colon, head and neck, renal carcinoma, acute myeloid leukemia, Hodgkin and large-cell lymphomas and pancreatic cancer [9,10], Notch is undoubtedly oncogenic. Moreover, high-level expression of Notch-1 and its ligand Jagged-1 is associated with poor prognosis in breast cancer, bladder cancer, leukemia, and prostate cancer [11-13]. However, the roles of Notch signaling in intrahepatic cholangiocarcinoma have not yet been characterized. Thus, in the present study, we explored the role of Notch1 expression, especially in relation to migration, in ICC.

## Methods

### Intrahepatic cholangiocarcinoma patient samples

Intrahepatic cholangiocarcinoma tissues were collected from five patients who underwent hepatectomy in our Hospital. None of the patients had received preoperative chemotherapy or radiotherapy. The five cholangiocarcinoma patients included 3 cases with infiltration of the surrounding tissue (such as the liver, portal vein, nerve, and pancreas) and 2 cases with regional lymph node metastasis. The specimens were obtained with written informed consent from all patients. The study was approved by the Committees for Ethical Review of Research involving Human Subjects in our Hospital.

### Cell culture

The human normal biliary epithelial cells established from histologically normal liver tissues obtained from five patients who underwent liver transection for metastatic tumors were gifts from Dr. Ludwik K Trejdosiewicz (University of Leeds, UK) [14]. The human cholangiocarcinoma cell lines QBC939, RBE, and ICC-9810 were obtained from ATCC and cultured in Ham’s F12 Medium supplemented with 10% FBS at 37°C in a humidified chamber containing 5% CO_2_.

### Antibodies

Antibodies against Notch-1, E-cadherin, Vimentin, F-actin and α-SMA were purchased from Santa Cruz Biotechnology, Inc. (Santa Cruz, CA, USA). The GAPDH antibody was purchased from Sigma-Aldrich (St. Louis, MO, USA).

### RNA extraction and reverse transcription-PCR

Total RNA was extracted using TRIzol reagent (Invitrogen, Carlsbad, CA, USA) according to the manufacturer’s protocol. cDNA was synthesized using TaqMan RT reagents (Applied Biosystems) following the manufacturer’s instructions. The primers for Notch1 and the glyceraldehyde 3-phosphate dehydrogenase (GAPDH) control were synthesized by Invitrogen (Carlsbad, CA, USA). The upstream Notch1 primer was 5′-GCAAGAAGAAGCGGAGAG-3′, and the downstream primer was 5′- AGCTGGCACCCTGATAGATG -3′; the Notch1 PCR product length was 423 bp. The upstream control GAPDH primer was 5′-AGATCCACAACGGATACATT-3′, and the downstream primer was 5′-TCCCTCAAGATTGTCAGCAA-3′; the GAPDH PCR product length was 308 bp. The PCR conditions were as follows: predenaturing at 94°C for 2 min, denaturing at 94°C for 30 s, reannealing at 53°C for 45 s, and elongation at 72°C for 30 s, for 30 cycles; and final elongation at 72°C for 10 min. The PCR products underwent 1.5% agarose gel electrophoresis.

### Western blot analysis

Protein was quantified using the Bradford assay (Bio-Rad, Hercules, CA, USA), and equal amounts of protein were separated on SDS-polyacrylamide gels and transferred onto nitrocellulose membranes (Amersham Biosciences, Piscataway, NJ, USA). After blocking in 5% skim milk for 1 h at room temperature, the membranes were incubated with the indicated primary antibody at 4°C overnight, followed by a horseradish peroxidase-conjugated secondary antibody. The proteins were detected by chemiluminescence (Amersham Biosciences, Piscataway, NJ, USA). The Western blot data were quantified by measuring the intensity of the hybridization signals using an image analysis program (Fluor-ChemTM 8900, Alpha Inotech).

### Plasmid constructs and siRNA transfection

The full-length Notch1 cDNA was amplified and cloned into the pReciever M68 expression vector (FulenGen, Guangzhou, China). The expression plasmids were transfected into cells using Lipofectamine 2000 (Invitrogen, Carlsbad, CA, USA) according to the manufacturer’s instructions.

Oligonucleotide siRNA duplexes were synthesized by Shanghai Gene Pharma (Shanghai, China). The following siRNA sequences for Notch1 were used: 5′- UGGCGGGAAGUGUGAAGCG-3′ and 5′- CGCUUCACACUUCCCGCCA-3′. The siRNAs were transfected into ICC-9810 cells with Lipofectamine 2000 (Invitrogen, Carlsbad, USA) according to manufacturer’s instructions.

### BrdU incorporation analysis

Ten micrograms per milliliter of BrdU were added to the culture medium for 24 h. The cells were fixed with 100% ethanol for 10 min, then incubated with 2 ml HCl for 45 min and 0.1 ml sodium tetraborate for 15 min at room temperature. The cells were then incubated with a mouse monoclonal anti-BrdU antibody overnight at 4°C and incubated with fluoresce in isothiocyanate-conjugated goat anti-mouse IgG for 1 h at room temperature. Hoechst 33342 was used to label nuclei.

### Rac activation assay

Rac1 intracellular activity was examined using Rac1 activation assay kits (Upstate Biotechnology, Lake Placid, NY, USA) according to the manufacturer’s protocols. Briefly, cells were lysed with Mg2+ lis buffer. After clarifying the cell lysates with glutathione agarose and quantifying the protein concentrations, aliquots with equal amounts of proteins were incubated with the Rac assay reagent (PAK-1 PBD, agarose) at 4°C for 1 h, using the GTPgS-pretreated lysates as positive controls. The precipitated GTP-bound Rac1 was then eluted in Laemmli reducing sample buffer, resolved by 12% SDS-PAGE, and immunoblotted with a monoclonal anti-Rac1 antibody. Five percent of the cell lysate was resolved by 10% SDS-PAGE and immunoblotted with a Rac1 antibody to measure the total amount of Rac1.

### Immunocytochemistry

Immunohistochemical staining was performed on 4-μm paraffin-embedded kidney sections. Antigen retrieval was performed by microwave treatment. The sections were exposed to 3% H_2_O_2_ for 20 min, blocked with 10% sheep serum in PBS at 37°C for 40 min, then incubated with the indicated antibodies at 4°C overnight. After rinsing three times with PBS, the sections were incubated with ChemMate™ EnVision/HRP Rabbit/Mouse secondary antibody (Dako, Copenhagen, Denmark) for 1 h. The degree of immunostaining was reviewed and independently scored by two observers based on the proportion of positively stained tumor cells and intensity of staining.

### Migration assay

Cells (1 × 10^5^) were suspended in 200 μl of serum-free DMEM medium and seeded on the upper side of the invasion chamber (Millipore, Billerica, USA). The lower side of the chamber was filled with DMEM supplemented with 10% fetal bovine serum. After incubation at 37°C for 18 h, cells that had penetrated through the chamber were fixed with methanol for 15 min at room temperature and stained with 0.1% crystal violet for another 15 min. The upper surface of the chamber was carefully wiped with a cotton-tipped applicator. Cells that had passed through the pores were counted in five non-overlapping fields (×40 magnification) and photographed.

### Cell morphology examination and immunofluorescence

Cell morphology was monitored on a phase contrast microscope equipped with a video camera. Cells grown on glass coverslips were fixed with 3.7% formaldehyde solution in PBS for 10 min at room temperature. Following three extensive washes with PBS, the cells were permeabilized in PBS containing 0.1% Triton X-100 for 3 min and blocked with PBS containing 5% BSA for 1 h at room temperature. The cells were incubated overnight at 4°C with primary antibodies diluted in PBS containing 3% BSA, followed by incubation with Alexa Fluor 488-conjugated goat anti–rabbit secondary antibody (1:1000; Molecular Probes, Eugene, OR, USA) for 1 h at room temperature for detection. Actin filaments were visualized by staining the cells with Alexa Fluor 633-conjugated Phalloidin (1:1000; Molecular Probes, Eugene, OR, USA) for 1 h at room temperature. To identify nuclei, the cells were counterstained with DAPI (Invitrogen, Carlsbad, CA, USA) for 3 min. The coverslips were mounted in fixation medium (Biomeda, Foster City, CA, USA). Images were collected and analyzed using the Zeiss LSM 510 Confocal Imaging System (Zeiss, Germany).

### Statistical analysis

The statistical analyses were performed using SPSS 13.0 statistical software (Chicago, IL, USA). Significant differences between two groups were determined by Student’s t-test. *P* < 0.05 was considered statistically significant. The results are expressed as the mean ± SD from at least three experiments.

## Results

### Notch1 was up-regulated in ICC tissues and cell lines

**Figure 1:**
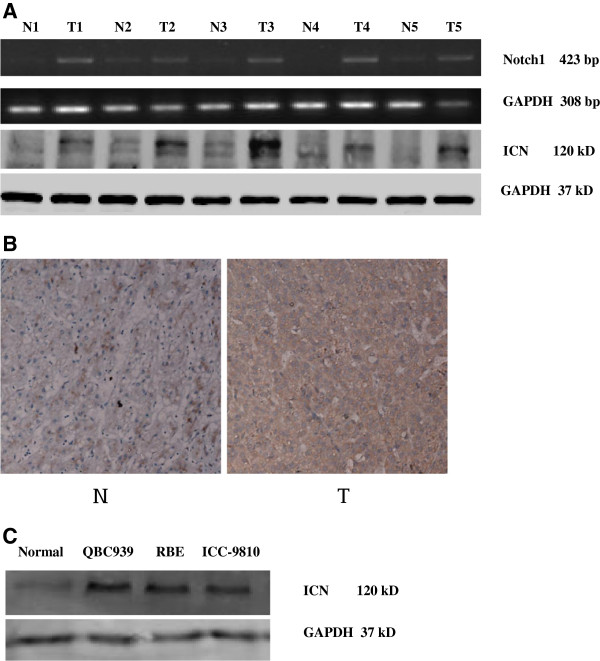
**Notch1 is up-regulated in ICC tissues and ICC cell lines. **(**A**) The expression of ICN (the intracellular domain of Notch1) is elevated in primary ICC tumors (T) compared with ICC tumor-adjacent tissues (N) examined by Western blotting. (**B**) The expression of Notch1 mRNA in each of the primary ICC tumor (T) and ICC noncancerous tissue (N) pairs from the same patient by reverse transcription-PCR. β-actin was used as a loading control. (**C**) The expression of ICN protein is elevated in ICC cell lines.

Abnormally high Notch1 expression has been implicated in many malignancies, but the pathological function of Notch1 in ICC has not been well defined. Therefore, reverse transcription-PCR and Western blotting analyses were performed on paired samples of ICC tissue and noncancerous tissue adjacent to the cancer lesion isolated from the same patient. Notch1 was found to be over expressed at both the mRNA and protein levels in all five ICC samples examined compared to adjacent tissue from the same patient (Figure 1A). Interestingly, among the five cholangiocarcinoma patients, patients No. 1, 2, and 3 displayed infiltration of the surrounding tissue (invasion of the liver, portal vein, nerve, and pancreas), and patients No. 2 and 3 displayed regional lymph node metastases.

We further investigated Notch1 protein expression in ICC specimens and normal control liver tissues using immunohistochemical analysis. Notch1 staining was primarily localized to the cell membrane and cytoplasm, suggesting that the protein was active. No significant Notch1 staining was observed in normal liver tissue; only weak staining was observed in the cell membrane and cytoplasm of a few cells (Figure 1B).

We next examined the expression of Notch1 in normal and ICC cells. As shown in Figure 1C, all cancer cell lines expressed high levels of Notch1 compared with normal biliary epithelial cells. The aberrant Notch1 expression in both ICC tissues and ICC cells suggests that increased Notch1 expression might be associated with tumor progression.

**Figure 2:**
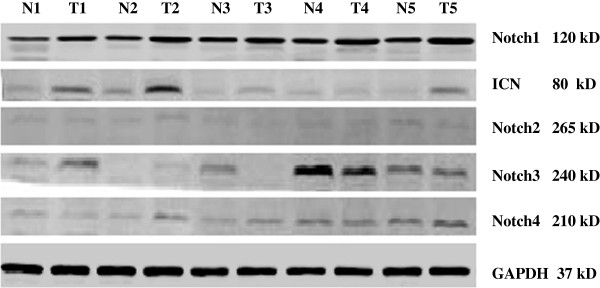
**The expression of other Notch receptors (Notch2, Notch3, and Notch4) in ICC tissue and noncancerous tissue adjacent to the cancer lesions. **Notch1 was found to be overexpressed in all five ICC cancer samples examined compared to normal adjacent tissue from the same patients, but the other receptors were not differentially expressed.

We also examined the expression of other Notch receptors (Notch2, Notch3, and Notch4) in ICC tissue and noncancerous tissue adjacent to the cancer lesions. As shown in Figure 2, Notch1 was found to be overexpressed in all five ICC cancer samples examined compared to normal adjacent tissue from the same patients, but the other receptors were not differentially expressed.

### Notch1 over expression activated Rac1 and promoted ICC cell migration

**Figure 3:**
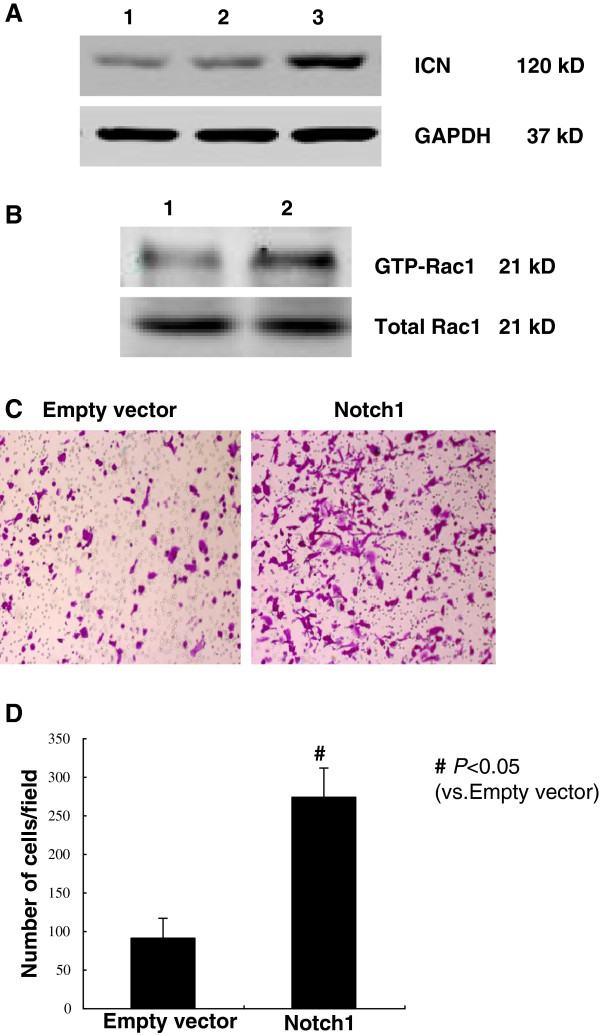
**Overexpression of Notch1 activated Rac1 and promoted cell migration. **(**A**) ICC-9810 cells were transfected with empty vector or Notch1, respectively. 48 h after transfection, the amount of active GTP-bound Rac1 was determined by antibodies specific for Rac1. (**B**) For Boyden chamber motility assay, cells were seeded onto the filter in the upper compartment of the chamber and incubated for 18 h. Cells passing through the pores were counted in five non-overlapping × 40 fields and photographed. *P < 0.05 vs vector transfected cells. (**C**) The activity of Rac1 after transfection 48 h. (**D**) The number of cells penetrated through the chamber in Migration assay. Notch1 transfected cells high expressed Notch1 compared with the cells in the control group. The number of cells penetrated through the chamber increased obviously. To prove that the increase of cells penetrated through the chamber was caused by the ability of cell migration, rather than cell proliferation, we also employed BrdU incorporation analysis to assay for cell proliferation. *P < 0.05 vs vector transfected cells. Data are expressed as mean ± SD of three independent experiments.

Exogenous expression of Notch1 in glioma cells has been shown to increase their migratory and invasive capacity [15]. To explore the function of Notch1 upregulation in ICC, exogenous Notch1 was transfected into ICC-9810 cells. We first examined Rac1 activity. As shown in Figures 3A, 3B, and 3D, over expression of Notch1 resulted in a dramatic increase in the GTP-loaded Rac1 compared with empty vector-transfected cells. Given the important role of Rac1 activation in cell migration, we next examined the effect of Notch1 over expression on cell migration using a Boyden chamber system (Figure 3C).

### Notch1 knockdown inactivated Rac1 and inhibited ICC cell migration

**Figure 4:**
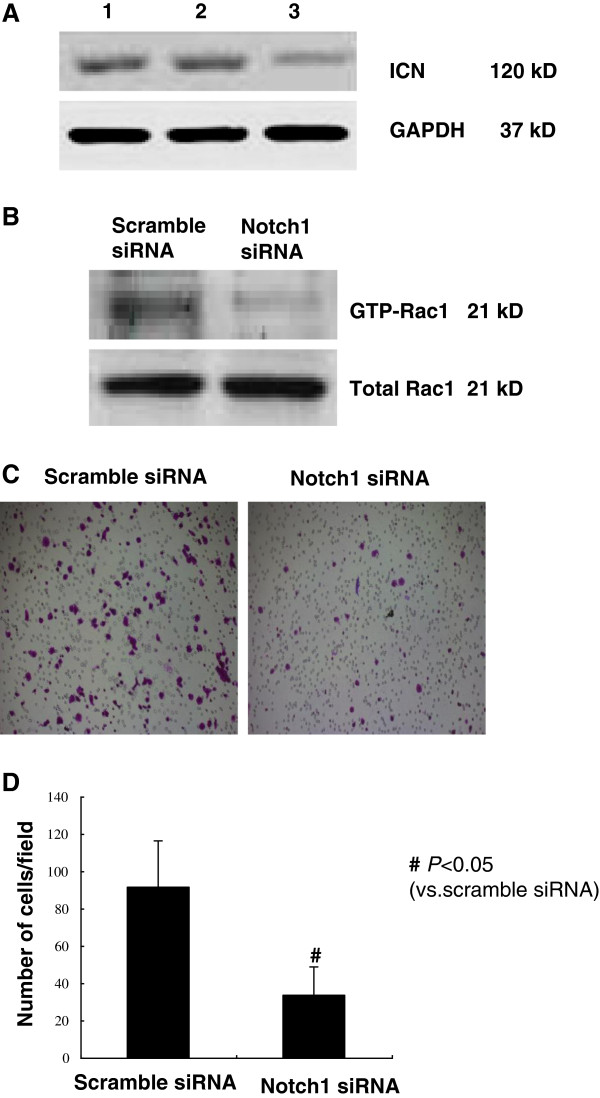
**Knockdown of endogenous Notch1 inhibits Rac1 activity and cell migration.** ICC-9810 cells were transfected with Notch1 siRNA or scramble siRNA (Control) (**A**). The ICN is sensitive to gamma-secretase inhibitor. Western blot analysis was employed to the ICN expression of ICC-9810 cells treatmented with 1 μmol/L of GSI. The results confirmed that ICN was induced at the protein level within 24 hours of exposure to the GSI but not by the DMSO control. Forty-eight hours after transfection, cell migration (**B** and **C**) and the Rac1 activity (**D**) were examined. *P < 0.05 vs. scramble siRNA-transfected cells. The data are expressed as the mean ± SD of three independent experiments.

In reciprocal experiments, we examined whether knocking down endogenous Notch1 would inhibit Rac1 activity and cell migration using Notch1 siRNA. The efficiency of the Notch1 siRNA was examined by Western blot (Figure 4A). Compared with scramble siRNA-transfected cells, the GTP-Rac1 level was dramatically decreased in Notch1 siRNA-transfected cells (Figure 4B). Similarly, suppression of Notch1 expression inhibited cell migration compared with scramble siRNA-transfected cells (Figure 4C). Knockdown of endogenous Notch1 inhibits Rac1 activity and cell migration. ICC-9810 cells were transfected with Notch1 siRNA or scramble siRNA (Control) (Figure 4D).

### Notch1 expression did not stimulate ICC cancer cell proliferation

**Figure 5:**
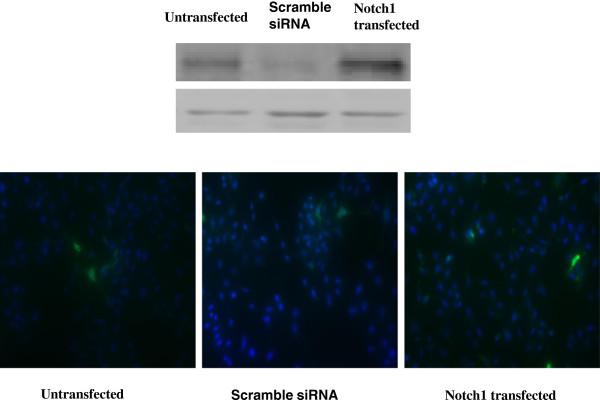
**Cell growth proliferation was assessed by BrdU incorporation analysis.** BrdU-positive cells, stained green. The number of positive cells reflects the ability of proliferation. However, no change of the number of BrdU -positive cells was observed in the Notch1 down- expression group or Notch1 high expression group compared to the control group. The results demonstrated that Notch1 down-regulating expression or high expression did not have any effect on cell proliferation.

To exclude the possibility that the increased cell migration was due to Rac1 activation, not cell proliferation, we over expressed and knocked down Notch1 in ICC-9810 cells, which express moderate levels of Notch1. As shown in Figure 5, BrdU incorporation analysis indicated that Notch1 expression did not affect cell proliferation.

### Notch1 over expression induced an EMT phenotype in ICC cancer cells

**Figure 6:**
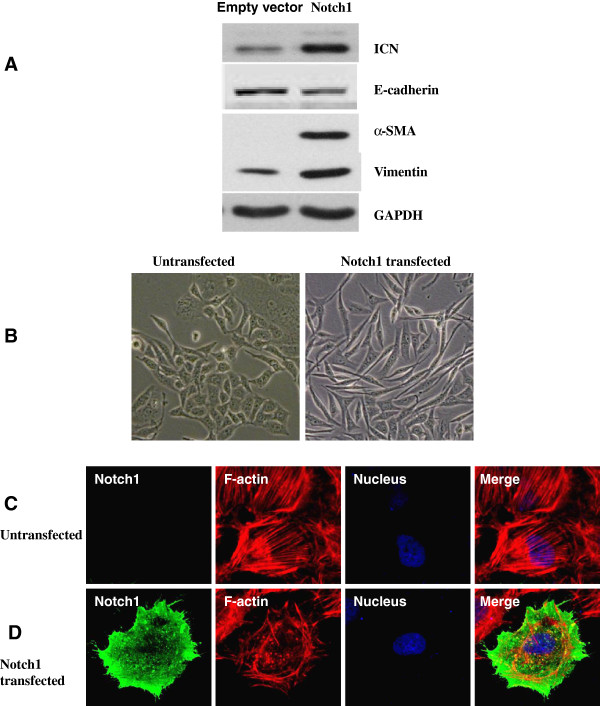
**Notch1 overexpression induces an EMT phenotype in ICC-9810 cells. **(**A**) Increased Notch1 expression was accompanied by enhanced expression of α-SMA and Vimentin and loss of E-cadherin expression, which are hallmarks of EMT. (**B**) Overexpression of Notch1 results in morphological changes in ICC-9810 cells. Photographs were taken using a Nikon microscope (phase contrast). Original magnification × 400. (**C **and **D**) ICC-9810 cells were infected with Notch1 or vector for 36 h before fixation and processing for immunofluorescence staining. Actin filaments were visualized by staining with Alexa Fluor 633-Phalloidin (red). Notch1 was visualized by staining with an anti-Notch1 antibody followed by Alexa Fluor 488-conjugated anti-rabbit IgG (green). Nuclei were stained with DAPI (blue). Images were taken by confocal microscopy. Original magnification × 630.

To demonstrate that the ICN is sensitive to gamma-secretase inhibitor, we performed western blot analysis to evaluate the ICN expression in ICC-9810 cells treated with 1 μmol/L GSI. This analysis confirmed that ICN was induced at the protein level within 24 hours of exposure to the GSI but was not induced by the DMSO control (Figure 6).

Notch activation has been shown to induce an epithelial to mesenchymal transition in breast cancer [16] Given that Notch1 expression was associated with ICC metastasis, we further investigated whether a link exists between Notch1 expression and the EMT phenomenon in ICC. Exogenous Notch1 was transfected into ICC-9810 cells that express moderate levels of Notch1. Increased Notch1 expression was accompanied by enhanced expression of α-SMA and Vimentin and loss of E-cadherin expression, which are hallmarks of EMT (Figure 6A). An examination of cell morphology and immunofluorescence indicated that Notch1 over expression resulted in morphological changes and cytoskeletal reorganization in ICC-9810 cells (Figure 6).

## Discussion

Notch genes encode large transmembrane proteins that act as receptors for the Delta, Serrate, Lag-2 (DSL) family of ligands [17]. Four different Notch proteins and the following five known ligands exist in mammals: Delta-like 1, Delta-like 3, Delta-like 4, Jagged 1 and Jagged 2 [18-22]. Notch signaling plays multiple roles in development and tissue homeostasis, and these roles can be subverted during oncogenic transformation. Despite the wealth of data suggesting a role for Notch in solid tumors, little evidence exists to support a causative role for Notch in tumor initiation in human solid cancers. Indeed, unlike in T-ALL, genetic alterations in Notch genes have not been identified in solid tumors. However, Notch signaling appears to be crucial in many solid tumors, including cancers of the breast, colon, pancreas, prostate and central nervous system [23]. Interestingly, Notch signaling also seems to play a contradictory tumor suppressor role in mouse keratinocytes, pancreatic and hepatocellular carcinoma, and small-cell lung cancer [24]. Taken together, these observations indicate that Notch exerts its effects in solid tumors as a result of aberrant activation of the pathway. Moreover, the cellular interpretation and outcome of aberrant Notch activity is highly dependent on contextual cues, such as interactions with the tumor microenvironment and crosstalk with other signaling pathways.

Intrahepatic cholangiocarcinoma is the second most prevalent intrahepatic primary cancer and has poor prognosis. The lethality of the disease is caused by both rapid tumor growth and the tendency to invade adjacent organs and metastasize [25].

Mounting evidence has demonstrated that EMT is associated with the invasive and migratory ability of cancer cells, conferring enhanced metastatic properties to these cells [26-28]. Increased expression of Notch1 has been shown to promote EMT in glioma; however, the role of Notch1 in ICC remains unclear.

In the present study, we found that Notch1 mRNA and ICN (the intracellular domain of Notch1) expression is higher in ICC tissue than in noncancerous tissue adjacent to the cancer lesions, and all cancer cell lines expressed high levels of ICN compared with normal biliary epithelial cells. Taken together, aberrant Notch1 expression in both ICC tissues and ICC cells suggests that increased Notch1 expression might be associated with tumor progression.

To elucidate the effects of Notch1 expression in ICC cells, separate over expression and knockdown experiments were conducted in ICC-9810 cells. Notch1 cDNA was introduced into ICC-9810 cells, and Notch1 protein expression was successfully induced. Notch1 over expression promoted migration and Rac1 activation in these cells. In contrast, the down-regulation of Notch1 inhibited the migration of ICC-9810 cells and resulted in dramatic decreases in Rac1 activity compared to control cells. Substantial evidence has indicated that increased Notch1 expression is accompanied by enhanced expression of α-SMA and Vimentin and loss of E-cadherin expression, which are hallmarks of EMT.

The Rho-like GTPase Rac1 is involved in migration and adhesion by modulating the actin cytoskeleton. Rac1 acts as a molecular switch, cycling between an active GTP-bound state and an inactive GDP-bound state, which is controlled by GEF. Rac1 is preferentially activated at the leading edge of migrating cells where it induces the formation of actin-rich lamellipodia protrusions that are thought to be a key driving force for membrane extension and cell movement. Rac1 is also an important regulator of the actin cytoskeletal dynamics that modulate cell migration and invasion [29]. Elevated levels of GTP-Rac1 have been shown to correlate with tumor metastasis and vascular endothelial growth factor (VEGF) expression [30]. Despite the importance of the upstream signaling mechanisms that facilitate Rac activation, the identity of these mechanisms in ICC remains unknown. In the present study, we demonstrated for the first time that the protein level of Notch1 is elevated in ICC tissues and that Notch1 over expression promotes migration and Rac1 activation in human ICC-9810 cells. By examining cell morphology and immunofluorescence, we found that Notch1 over expression results in morphological changes and alterations in the F-actin cytoskeleton in ICC-9810 cells (Figure 4). These results suggest that upregulation of Notch1 could promote ICC cell migration and invasion through Rac1 activation.

In the present study, Rac1 inhibition attenuated the effects of γ-secretase on Notch1, resulting in decreased production of the Notch1 intracellular domain and a slight decrease in the shedding of the ectodomain form of Notch1 [31]. We have shown that down-regulation of Notch1 results in a dramatic decrease in Rac1 activity, suggesting that a mechanism exists to determine whether Rac1 or Notch1 is the preferred substrate for γ-secretase; however, this mechanism requires further elucidation.

## Conclusions

In conclusion, we have shown here that Notch1 expression is upregulated in clinical ICC specimens and promotes tumor migration, indicating that Notch1 may be involved in ICC carcinogenesis and progression. These findings suggest that Notch1 could serve as a novel diagnostic and therapeutic target in patients with ICC and thereby establish the potential for targeting Notch signaling as an approach to inhibit tumor metastasis.

## Competing interests

The authors declare no competing interest.

## Authors’ contributions

QZ and JL have made substantial contributions to design of project and acquisition of data. QZ and YW performed experiments. BP, LL have made substantial contributions to analysis and interpretation of data. JL and YW wrote manuscript. JL has given final approval of the version to be published. All authors read and approved the final manuscript.

## Pre-publication history

The pre-publication history for this paper can be accessed here:

http://www.biomedcentral.com/1471-2407/13/244/prepub
